# Examination of Blood-Stains

**Published:** 1868-05

**Authors:** 


### ARTICLE IV.

### *Examination of Blood-Stains.*

Dr. Thomas Price, Professor of Chemistry, Toland Medical College, has the following communication in the Pacific Medical and Surgical Journal:

On the 28th of February last, the clothes of an Indian who it was suspected had murdered an old woman of seventy years of age, at Martinez, Contra Costa county, were brought to me, in order that I might subject the blood-stains found on them to a chemical and microscopical examination. The evidence against the prisoner was entirely circumstantial, and created great interest during the whole trial. The Hon. J. W. Dwinelle, the presiding judge remarked that he had never sat upon a more interesting case. The evidence, although entirely circumstancial, was overwhelming; and, as the able and indefatigable prosecuting attorney, H. Mills, remarked, "formed a complete, and perfect chain, without a single link missing." When the Indian was examined, at first, and before being committed to jail, he accounted for the blood-stains found on his clothes, by his having helped a lady to kill a turkey a few days before. This statement rendered my examination a very easy one. Had he contended that he had been assisting to slaughter some of the domestic mammalia, the science of chemistry and microscropy would have failed in adding a link to the chain of evidence against him, especially that the stains were some thirty days old when the clothes were handed over to me. The following is a description of the method I adopted in my examinations, to prove that the stains were really blood:

1. By Chemical tests. Portions of the smeared clothes were digested in water, alkaline salts, and glycerine, which yielded me, after several hours' soaking, a solution having the characteristic red color of blood, which suffered no change on addition of ammonia, and coagulated on boiling and on addition of nitric acid. These chemical tests were only used to establish the fact that the stains were, or were not, due to blood. So far we are not able to find any distinct difference between the blood of man and that of any other animal by chemical tests; yet it was contended at one time, by specialists in this department, that they could discover, by the smell of the blood, to what animal it belonged ; and possibly one may so educate his nose as to be able to form an opinion, and probably the blood of a goat may be pronounced upon with certainty by the odor ; but it is my experience, that it is better not to rely upon the odor.

2. By microscopical examination. The red-colored liquor obtained above, presented, under the microscope, the well-known blood globules, in the form of circular flattened discs. This fact, connected with the chemical tests, proved that the spots were due to blood ; and more than that, the form of the corpuscles showed conclusively, that it was the blood of one of the *mammalia*. The micrometer was now attached to the microscope, and the corpuscles measured, with the following result: size, 1-2000 of an inch to 1-4000 of an inch; average, 1-3200 of an inch ; from which result it was possible that the blood could be human blood, as these measurements are the same as those given by eminent microscopists as the size of man's blood. And they agreed very nearly with measurements of the globules of my own blood, that of my assistants, and others ; but from the fact that the stains were old, I could not state positively that the blood I found on the clothes was that of a human being. I could, and did state, that it was not the blood of a turkey, and that it must be the blood of one of the mammalia. The size of the corpuscles corresponded with those of man. I could not swear that it was human blood ; nor could I, on the other hand, state that it was not. Since there are no means of ascertaining that the dried globules have assumed their original size, their measurement affords no positive proof that they are from the blood of a man. At this stage of my examination, and at the request of Judge Dwinelle, I telegraphed to San Francisco for my microscope, as some of the jurors had made up their minds that it was impossible to detect any difference between the blood of the various animals, birds, fishes, and frogs. They could see no difference with their own eyes ; and never having looked through a powerful microscope, they thought it purely imaginary. The jury were shown under the microscope, the following bloods: that of a frog, chicken, turkey, hog, calf, cow, dog, and horse, and each one compared with blood from my own finger. The whole jury were much interested in these experiments; and the most skeptical of them confessed that the difference between the blood of the turkey, chicken, and frog, was so great, that it was impossible to mistake one for the other. Some of them even went so far as to say, that they could distinguish with their eyes, and, without the use of a micrometer, detect the difference between the blood of a hog, cow, and calf, and my blood. All of them were now entirely convinced of the value and reliability of the microscope. The jury found the Indian guilty, and on the last day of July he suffered the extreme penalty of the law.

